# Involvement of Arsenic Atom of AsF_3_ in Five Pnicogen Bonds: Differences between X-ray Structure and Theoretical Models

**DOI:** 10.3390/molecules27196486

**Published:** 2022-10-01

**Authors:** Steve Scheiner, Mariusz Michalczyk, Wiktor Zierkiewicz

**Affiliations:** 1 Department of Chemistry and Biochemistry, Utah State University, Logan, UT 84322-0300, USA; 2 Faculty of Chemistry, Wrocław University of Science and Technology, Wybrzeże Wyspiańskiego 27, 50-370 Wrocław, Poland

**Keywords:** pnicogen bond, cooperativity, cluster, AIM, NBO

## Abstract

Bonding within the AsF_3_ crystal is analyzed via quantum chemical methods so as to identify and quantify the pnicogen bonds that are present. The structure of a finite crystal segment containing nine molecules is compared with that of a fully optimized cluster of the same size. The geometries are qualitatively different, with a much larger binding energy within the optimized nonamer. Although the total interaction energy of a central unit with the remaining peripheral molecules is comparable for the two structures, the binding of the peripherals with one another is far larger in the optimized cluster. This distinction of much stronger total binding within the optimized cluster is not limited to the nonamer but repeats itself for smaller aggregates as well. The average binding energy of the cluster rises quickly with size, asymptotically approaching a value nearly triple that of the dimer.

## 1. Introduction

The pnicogen bond (PnB) is closely related to the H-bond in a number of respects. A PnB occurs when the bridging H of an H-bond is replaced by any of the pnicogen family, e.g., P, As, Sb, or Bi. The Pn atom is usually trivalent and each of the PnR_3_ bonds draws electron density toward itself, leaving a deficiency along the extension of this bond near the Pn center. This reduced density, commonly referred to as a σ-hole, leads in turn to a localized region of positive electrostatic potential which can attract a nucleophile. In addition to the associated coulombic attraction, another major contributor to the stability of the PnB is due to a certain amount of charge transfer from the nucleophile to an antibonding σ*(PnR) orbital, which is itself generally coincident with the σ-hole. The final component of the PnB is the dispersive attraction that is present.

The general framework of the PnB is repeated in a number of very similar noncovalent bonds, known as halogen, chalcogen, tetrel, and triel bonds [1,2,3,4,5,6,7,8,9,10], depending upon the family of elements from which the bridging atom is drawn.

The PnB has gained increasing attention in the literature and has made its presence felt in a number of different environments. It has been observed in a varied set of crystals [11,12,13,14,15,16], and in the gas phase [17]. Experimental observations have been made in matrices [18,19,20,21] and via NMR measurements [22,23], and there are some experimental estimates of PnB energies [24] available. PnBs are involved in numerous chemical functions such as the capture and transport of halides across a phospholipid membrane [25]. They can reverse the energetic balance between the chair and twist-boat conformations of cyclohexane [26]. The PnB is often involved in catalysis [27,28]: some examples include a chiral scaffold [29], enantioselective transfer hydrogenation of benzoxazines [30], and polyether cascade cyclizations [31]. Applications have been studied in terms of halide binding [32,33,34,35] and as a common factor in biological contexts [36,37] such as proteins and nucleic acids [38].

In addition to recent general reviews [39,40] of these bonds, where they occur, and how they influence chemical processes, there has been a good deal of scrutiny from the perspective of quantum calculations [41,42,43,44,45,46,47,48,49,50,51,52,53,54,55,56,57,58], which have led to a number of overarching conclusions. The strength of a PnB rises along with the size of the Pn atom. As the lightest member of this family, N is typically not involved as an electron acceptor, although some exceptions do seem to occur [40,59,60,61,62,63,64,65]. The presence of electron-withdrawing substituents on the Pn atom strengthens the PnB, as does a more nucleophilic basic partner. The localization of the σ-hole and the σ*(PnR) antibonding orbital impart a high degree of sensitivity to angular distortion [66], even more so than the H-bond. There is also the possibility that a π-hole situated above a Pn atom can replace the more common σ-hole [67,68] and PnBs can also occur for a hypervalent Pn atom [21,54,56,69,70].

Although there is a myriad of crystals in which one or more PnBs have been identified, it usually is just one of several stabilizing interactions that are present. Given the potential strength of the PnB, there is a surprising dearth of crystals in which this type of bond serves as the driving force. It was therefore gratifying to note a very recent analysis of diffraction data for the crystal of AsF_3_ by Varadwaj et al. [71] in which the various molecules incontrovertibly engage in PnBs with one another, to the exclusion of any other factors which are present in other crystals. The data supplied in this paper offer a unique opportunity to study this noncovalent bond within a crystal environment, where it is not perturbed by the presence of other sorts of bonding interactions. By employing modern quantum chemical methods, it is possible to examine how the forces present in the extended crystal environment might differ from those in a smaller cluster of *n* molecules. In other words, by optimizing the structure of a progressively larger cluster, one can assess at what size the cluster assumes the geometry within a crystal. By comparison of the differences between the details of the crystal and cluster structures at each stage of theoretically predicted growth, one can better understand how the forces present within a fully extensive crystal shape its ultimate geometry. As another benefit of this particular system, as described below, AsF_3_ contains well-defined maxima and minima on its surrounding electrostatic potential, ideal for the study of pnicogen bonds connecting the individual units.

## 2. Computational Details

Solid-state geometries were accessed through the Cambridge Structural Database (CSD, ver. 5.42 with updates) and supporting CCDC software Mercury and ConQuest [72,73]. The original supercell crystal structure of arsenic trifluoride (AsF_3_) was reported earlier [74] and used in this work as the starting point for further considerations. Quantum calculations were performed at the PBE0-D3/def2TZVP [75,76,77] level of theory. Calculations of crystal segments invoked structures from the CSD directly with no optimization, whereas cluster structures were optimized as described below. The difference in energy between a given cluster and its molecular components, taken in their geometries within the cluster, was defined as the interaction energy. Basis set superposition error (BSSE) was corrected via the counterpoise procedure introduced by Boys and Bernardi [78]. The quantum calculations were carried out within the framework of the Gaussian 16, Rev. C.01 set of codes [79]. Bader’s QTAIM protocol [80] provided analysis of the electron density topology by means of the AIMAll suite of programs [81] and NBO characterization was carried out within the natural bond orbital (NBO) framework [82,83]. Non-covalent interaction (NCI) description of the reduced density gradient made use of the Multiwfn program and visualized through VMD [84,85]. The extrema of the electrostatic potentials were identified and quantified via Multiwfn.

## 3. Results

**Figure 1:**
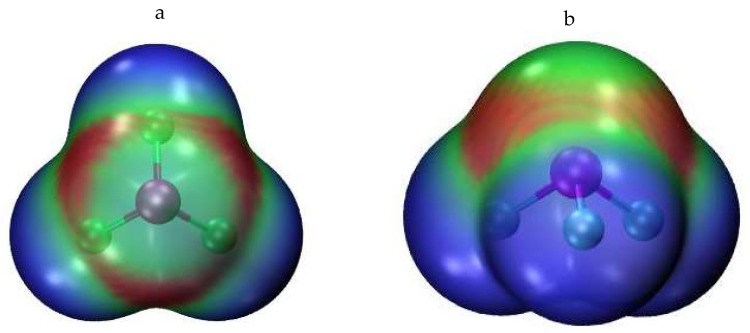
Molecular electrostatic potential of AsF_3_ on isodensity surface corresponding to 0.001 au (**a**: top and **b**: side views). Red regions are most positive (38.0 kcal/mol) and blue the most negative (−15.9 kcal/mol).

As an initial probe of the building block, the molecular electrostatic potential (MEP) of the optimized AsF_3_ monomer was evaluated. As is evident in Figure 1, there are three σ-holes present on the As, each with an MEP maximum of 38.0 kcal/mol. The As lone pair pushes them 14° away from the corresponding F-As projection, so the θ(F-As-V_s,max_) angle is 166°. The minimum located on each of the three F atoms amounts to −15.9 kcal/mol. It lies roughly along one of the F lone pairs, with a θ(As-F-V_s,min_) angle of 142°. With these well-defined maxima and minima, and of substantial magnitude, the AsF_3_ molecule serves as an ideal prototype by which to examine the pnicogen bonds within the aggregates. Indeed, the interaction energy of the PnB within the simple AsF_3_ dimer amounts to 4.4 kcal/mol (see below), competitive with the strength of the ubiquitous H-bond that is the basis of innumerable crystals.

### 3.1. Nonamers

**Figure 2:**
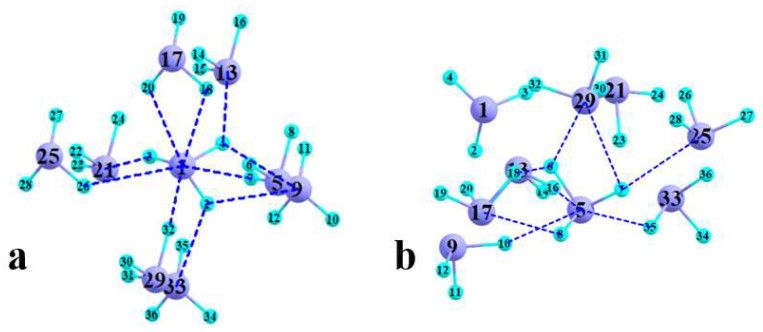
Geometries of nonamer taken from (**a**) X-ray structure of crystal and (**b**) optimization. Light blue refers to F atoms and As shown in purple. Dashed lines indicate AIM bond paths involving central AsF_3_ unit.

**Figure 3:**
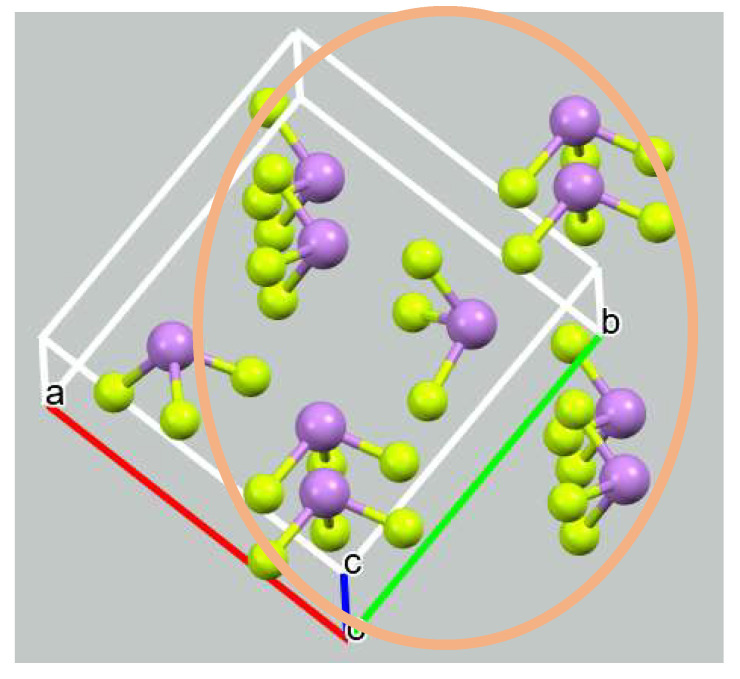
Arrangement of AsF_3_ nonamer (in brown ellipse) in relation to unit crystal cell (purple ball color indicates an arsenic atom and celadon ball color indicates a fluorine atom).

Examination of the crystal structure offers a picture wherein each AsF_3_ unit is surrounded by eight nearest neighbors. The geometry of this nonamer is depicted in Figure 2a whereas its arrangement versus the unit cell is shown in Figure 3, and broken blue lines indicate where AIM places interatomic bond paths. Five such bond paths invoke the As atom designated As1 and an F of four of the peripheral units. The R(As··F) distances in these polyfurcated bonds are listed in the first five rows of Table 1 and vary from 2.89 to 3.44 Å.

The density of each bond critical point is displayed in the next column of the table. This quantity grows as the interatomic distance becomes shorter, varying from 0.004 au for the longest such bond of 3.44 Å, up to 0.011 au for the two shortest bonds, less than 3 Å. The three F atoms of the central AsF_3_ all participate in bonds as well, each to different As atoms of the peripheral units. These bond lengths are in a similar range, as are the bond critical point densities. As a very crude estimate of the total bond strength involving the central unit, the sum of all ten densities in the last row of Table 1 comes to 0.0839 au.

As an alternative to the crystal geometry, the nonamer was built up by starting with an optimized dimer. As each additional AsF_3_ unit was added, the geometry of the trimer, tetramer, etc. was reoptimized, building upon the preceding structure with *n* − 1. The geometry of the final nonamer is pictured in Figure 2b and seems rather different in overall shape than the X-ray structure. As one distinction, there is no obvious central molecule in Figure 2b. However, the unit with the As5 atom most closely takes on this role, so its bond paths are indicated again by broken blue lines. The bond lengths listed in Table 1 are in the same general range of 2.86–3.46 Å as in the X-ray geometry. The same can be said of the AIM bond critical point densities, which again correlate nicely with the interatomic distances. It might be noted that one of the BCPs connects a pair of F atoms of two different molecules; more will be said about this issue below.

The total of all BCP densities in the cluster is 0.0607 au, a bit smaller than the 0.0839 au of the crystal geometry. However, this difference belies the actual energetics to some extent. The first row of Table 2 contains the sum of all pairwise interaction energies involving the central unit. There are eight such pairs, each computed using the geometry within the nonamer. This total of 13.9 kcal/mol is only slightly smaller than the interaction energy of 15.3 kcal/mol between the central molecule and the eight surrounding units considered as a single octameric entity, suggesting only a relatively small degree of cooperativity and higher-body interactions. The sum of pairwise interaction energies that involve the central As5F_3_ unit within the optimized cluster is slightly larger than that in the X-ray geometry, 15.8 vs 13.9 kcal/mol. One can conclude that there are only minor differences between the crystal and cluster binding energetics with respect to the central molecule.

**Figure 4:**
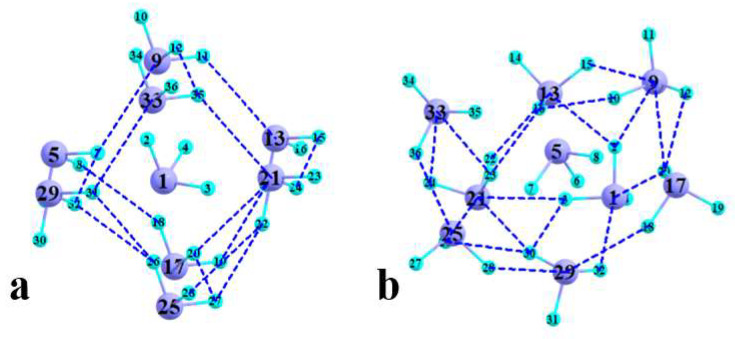
AIM bond paths involving only peripheral AsF_3_ units in (**a**) X-ray structure and (**b**) fully optimized cluster.

The most dramatic distinction between the crystal and cluster binding pattern involves the interactions between peripheral units, i.e., those that do not involve the central molecule. The numerous AIM bond paths for these two geometries are depicted in Figure 4a and Figure 4b, respectively. It is difficult to derive much quantitative information from these diagrams, except that there are a few more such bond paths in the cluster, 15 bonds for the former, as compared to 19 for the latter. The interatomic distance of each such bond path, and the density at its BCP, are reported in Table 3, which shows some clear differences. In the first place, these bonds are shorter in the optimized cluster: the minimum interatomic distance of the cluster is 2.915 Å as compared with 3.227 Å within the X-ray structure. The average distances of 3.108 and 3.396 Å, respectively, show a similar distinction. The shorter distances in the cluster are reflected in generally larger BCP densities: 0.0071 and 0.0028 au for the cluster and X-ray geometry, respectively.

The larger number of peripheral bonds in the cluster, in conjunction with their greater implicit strength, leads to a much larger sum of ρ_BCP_. This sum, amounting to 0.1357 au in the cluster, is more than three times larger than the 0.0423 au sum in the X-ray conformation. It is therefore no surprise that the total pairwise interaction energies involving only peripheral molecules, i.e., excluding the central unit is much larger in the cluster. As may be seen in Table 2, this peripheral pairwise sum is 35.7 kcal/mol in the cluster, as compared with only 6.2 kcal/mol in the X-ray geometry. When added together with the pairwise sums in the previous row that include the central molecule, the total cumulative pairwise energies in the cluster and X-ray structures are 51.45 and 20.14 kcal/mol, respectively. These quantities are not far removed from the total interaction energy within the entire nonamer in the next row of Table 2, arising from assembling the entire complex from nine separate monomers. This similarity indicates that the non-pairwise and cooperative effects are not overly large. In fact, what there is of such an effect favors the cluster whose total interaction energy is magnified by some 6% by their incorporation.

### 3.2. Nature of Bonding

**Figure 5:**
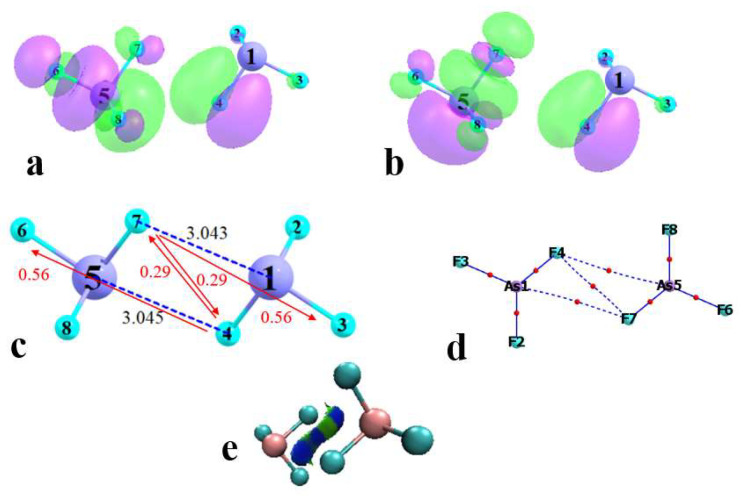
NBO orbital diagrams showing overlap of F4 lone pair with (**a**) σ*(As5F6) and (**b**) σ*(As5F7) in optimized AsF_3_ dimer (purple and green colors in (**a**) and (**b**) indicate opposite sign of wavefunction). (**c**) NBO values of E2 (kcal/mol) for transfer involving atoms denoted by red arrows. Interatomic distances in Å. (**d**) AIM diagram with bond critical points indicated as red dots. (**e**) NCI diagram of RDG with attractive regions indicated in blue, less attractive in green. RDG isovalue is 0.5 au.

As is apparent in the forgoing figures and tables, the bulk of the bond paths in these nonamers connects an As atom with an F on a neighboring molecule. Each such bond can be conveniently characterized as a classical pnicogen bond (PnB). The F atom in question lies roughly along the extension of an F-As covalent bond, which would place this F in proximity to the σ-hole on the surface of the As. As a second integral component of this sort of PnB, an F lone pair is well-aligned so as to donate a certain amount of electron density into the σ*(As-F) antibonding orbital. This orbital overlap is apparent in Figure 5a which illustrates the relevant NBO localized orbitals in the optimized geometry of the AsF_3_ dimer. The green lobe of the F4 lone pair on the right overlaps with the green lobe of the σ*(As5-F6) antibonding orbital; the ensuing charge transfer accounts for a second-order perturbation energy E2 of 0.43 kcal/mol. However, the forgoing is not the only opportunity for a charge transfer occasioned by orbital overlap. The same F4 lone pair can also interact with another σ*(AsF) orbital, this one involving F7 as depicted in Figure 5b. This overlap is not as perfect as that in Figure 5a, so E2 for this transfer is reduced to 0.21 kcal/mol.

The full picture of all the relevant intermolecular transfers are contained in Figure 5c where each red arrow indicates the direction of the transfer between the two atoms in question. The total transfer from F4 to the As5F6 antibonding orbital is 0.56 kcal/mol, marked by the red number on that arrow. This 0.56 arises from the 0.43 kcal/mol mentioned above, plus a small addition due to a second F4 lone pair. Likewise, the 0.21 kcal/mol transfer to the As5F7 antibonding orbital is supplemented by a small contribution from another F4 lone pair for a total of 0.29 kcal/mol. Another important point is that due to the symmetry of the dimer, there is an equal transfer from the F7 lone pairs to As1F3 and As1F4, accounting for the second set of arrows in the opposite direction. This equal and opposite transfer in both directions augments the strength of the interaction through cooperativity.

This brings us to the AIM diagram of this dimer. As displayed in Figure 5d, there are bond paths present between the As1-F7 and As5-F4 pairs, in accord with expectations of a typical pnicogen bond, as these F atoms line up with the σ-holes due to F3 and F6, respectively. But there is also a path connecting F4 and F7. In the absence of a proper understanding of the charge transfers described above, one might jump to the false conclusion of a halogen bond between these two F atoms. But in fact, this interaction is more correctly considered as a secondary component of the same pnicogen bonds, involving transfer to antibonding orbitals of AsF bonds other than that directly opposite the electron donor F. Indeed, the density of the F··F critical point is 0.0009 au, the same as the two As··F BCPs, so this supplement is far from negligible. The NCI diagram of this dimer in Figure 5e is consistent with this interpretation, displaying a blue attractive region, encompassing the two As··F axes as well as that between the two F atoms. In summary, then, the F··F AIM bond paths are part and parcel of the pnicogen bonds between molecules.

### 3.3. Building of Clusters

The foregoing analysis has focused on the fully developed nonamers, whether as a segment of the X-ray structure or as a fully optimized cluster. It would be enlightening as well to monitor the building of each aggregate in stages, adding one molecule at a time to a growing collection. The energetics of each size aggregate is contained in Table 4. It is immediately clear that the optimized clusters are considerably more strongly bound than the same-size oligomers extracted from the crystal. This advantage, in favor of the clusters, increases along with the size of the aggregate.

**Figure 6:**
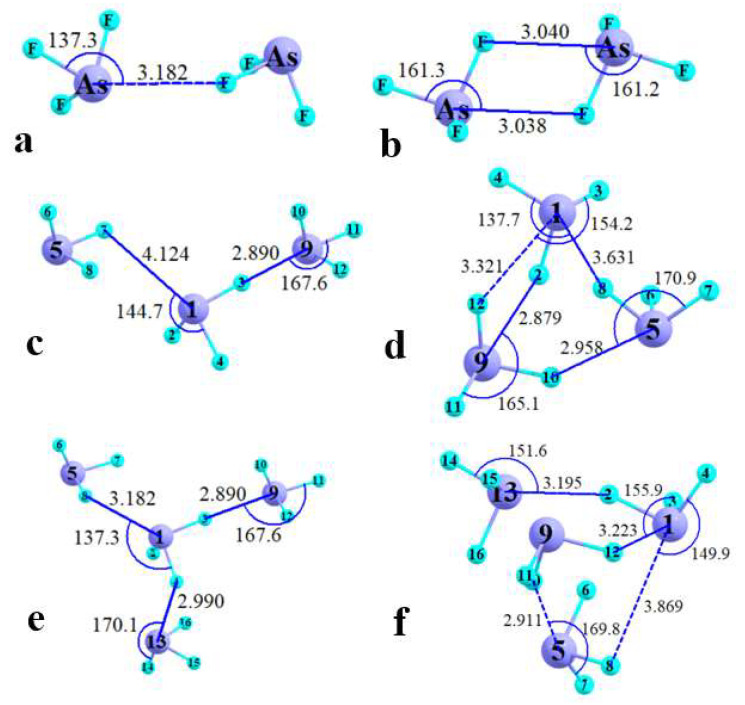
Geometries of *n*-mers of AsF_3_ taken from crystal on the left (**a**,**c**,**e**) and from optimization on the right (**b**,**d**,**f**). Distances in Å, angles in degrees.

Taking the dimer as a starting point, the reason for the better binding in the case of the optimized geometry is evident by a comparison of Figure 6a with 6b. There is one clear PnB present in the crystal dimer segment, with an R(As··F) distance of 3.18 Å, which compares with a pair of such bonds in the optimized dimer of Figure 6b, both with a shorter distance and F-As··F angles closer to linearity. The trimer segment of the crystal in Figure 6c contains two PnBs but one is highly stretched to 4.12 Å. The optimized trimer shown in Figure 6d is cyclic with four PnBs, two of which are shorter than 3 Å. It is thus no surprise that the interaction energy of the optimized trimer is more than twice that of the crystal geometry. Similar considerations apply to the tetramers in Figure 6e,f where the optimized geometry contains a larger number of shorter PnBs. As the clusters grow in size, the forgoing issues remain, and the optimized geometries are much more stable than the corresponding crystal extracts. In quantitative terms, the ratio between their total interaction energies lies between 2 and 3, averaging about 2.5.

**Figure 7:**
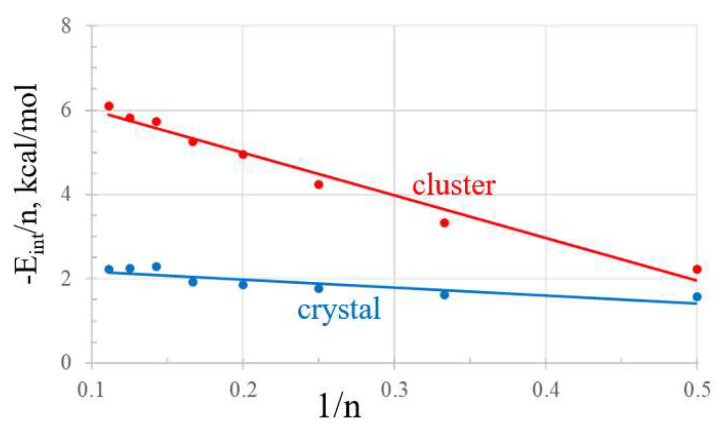
Variation of average interaction energy of crystal and cluster aggregates with reciprocal of size.

It is also worthwhile to consider the degree of cooperativity within each sort of aggregate. This aspect can be explored via the mean interaction energy of each aggregate, equal to the total interaction energy divided by the number of molecules present. This mean quantity is reported in the last two columns of Table 4 which exhibit an interesting trend. The average binding energy for the cluster grows continuously with *n*, reaching up to 6.1 kcal/mol for the nonamer. In fact, the red line in Figure 7 shows how this average energy scales linearly with 1/n, with correlation coefficient R^2^ = 0.98. Projection of this line to an infinitely large cluster would predict mean interaction energy of 7.0 kcal/mol, more than triple the PnB energy of the simple dimer. The behavior of the crystal segments is quite different. After an initial rise, E_int_/*n* plateaus at *n* = 7, even showing a small decline for further enlargement. The average of even the large aggregates is only slightly larger than the dimer energy.

### 3.4. Maximum Number of Pnicogen Bonds

**Figure 8:**
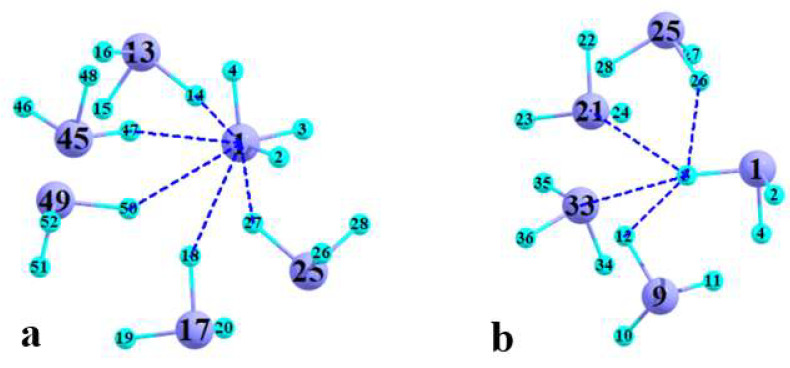
Bond paths involving (**a**) As1 and (**b**) F3 atom of central AsF_3_ unit within 14-mer extracted from crystal coordinates.

With three F substituents, it is logical to anticipate that AsF_3_ ought to contain three σ-holes, which would enable it to engage in three As··F PnBs. In order to test this idea, a central AsF_3_ molecule was more completely surrounded by 13 neighboring molecules, again within the context of the crystal structure. An AIM diagram of this 14-mer led to five separate bond paths from the As of this central molecule. These paths are illustrated in Figure 8a, and their geometric characteristics are listed in the upper portion of Table 5. Two of these PnBs, those to F14 and F47, are classic bonds in that the F of the electron donor lies within less than 20° of an F-As bond extension, i.e., near a σ-hole at the As atom. As indicated in the next column of Table 5, the densities of their BCPs are both over 0.01 au, in the range expected for a PnB of moderate strength.

However, the other three PnBs do not fall into this category, with θ(F-As··F) angles between 132° and 140°. The BCP densities of these three latter nonlinear arrangements are below 0.01 au, but not necessarily small, suggesting that the misalignment of the primary σ*(F-As) antibonding orbital by a small angle can be countered by transfer into the antibonding orbitals of the other two As-F bonds, as for example in the case described in Figure 5b.

As a further test of the PnB nature of these interactions, NBO analysis sought transfers from the F lone pairs to the corresponding σ*(AsF) antibonding orbitals. The cumulative sum of the E2 values, considering all three such σ*(AsF), is listed in the last column of Table 5. This sum exceeds 2 kcal/mol for the more linear arrangements in the first two rows and is between 0.5 and 1.0 kcal/mol for the next three that are less linear. These cumulative E2 quantities are roughly proportional to the AIM densities in the preceding column. Although some of these PnBs are rather weak, there do appear to be five such bonds to As1.

In a converse sense, it is also of interest to determine the number of PnBs in which an F atom can participate as an electron donor. Focusing again on the central AsF_3_ unit of the aforementioned 14-mer, the F3 atom participates in four separate bond paths to neighboring molecules, as exhibited in Figure 8b. Two of these paths lead to As atoms, one of them close to linear which has a fairly large density. The other two paths lead to another F atom, which as explained above does not disqualify them as a subtype of pnicogen bond. In fact, detailed NBO analysis indicates that the F3–F26 bond path represents a supplement to the As1–F27 PnB. The F3–F12 path, on the other hand, is not supported by NBO charge transfers and is fairly long, and thus may be an artifact of the AIM protocol.

## 4. Discussion

There is a clear difference between the structures adopted by a crystal with its enormous number of molecular units, and a cluster of limited size. The immediate neighbors of a single central unit within the crystal line up in an orderly and symmetric fashion, with a number of PnBs to it. There are also PnBs present in the cluster of size 9, although the structure of this nonamer does not place any one unit clearly in its center. Despite the difference in overall structure, the two geometries share similar total interaction energy of the central unit with its neighbors. Where the two geometries most differ has to do with the eight peripheral units. In the nonamer cluster, these units engage in numerous strong PnBs with one another. In fact, the cumulative interaction energy between these peripheral units is more than twice the interaction of the central unit with them. In the crystal geometry, by contrast, there are fewer interactions between peripheral units, and those bonds that are present are longer and on the weak end of the PnB spectrum. The sum of all of these interactions between peripheral molecules is less than half of that with the central unit. Taken together, the nonamer cluster is bound much more strongly than the aggregate of the same size within the context of the crystal geometry, 55 to 20 kcal/mol, respectively.

This distinction is not limited merely to the particular size of *n* = 9. At each stage of building the cluster, from *n* = 3 to *n* = 8, the binding energy of the crystal framework is less than half that in the fully optimized cluster. The average binding energy within the crystal context grows only slightly larger than the dimer with the larger size and has reached what appears to be its apex at *n* = 7. In contrast, the cluster arrangement shows a continuing trend of rising average interaction energy that shows no sign of abatement. A projection to infinite size would yield a mean interaction energy more than three times larger than the PnB energy within a dimer.

The foregoing leads to the central question as to why the crystal nonamer, and its smaller counterparts, are bound so much more loosely than they might otherwise be. The answer likely lies in the observation that the crystal segment and a like-sized cluster are similar in terms of the binding energy of a central molecule to its neighbors. Thus, it is reasonable to conclude that the various units in a crystal are well-disposed to form strong PnBs to a central molecule. The difference arises in the comparison of the bonding of the peripheral molecules with one another. The associated cumulative interaction energy is not very large in the crystal segment, only about 37% of what it is in a fully optimized cluster of the same size. In the cluster, these peripheral units have an incentive to engage in strong PnBs with one another as a means of stabilizing the entire system, and they adjust their positions accordingly. In a crystal, by contrast, the peripheral molecules have an alternative. Instead of bonding strongly with another neighbor of the central unit, they can bind with other molecules in the crystal, those that are more distant from the central unit, not included within the nonamer, or in any cluster of comparable size. It is for this reason that the internal geometry of any given cluster will differ appreciably from what is observed in a crystal.

Similar arguments apply to considerations of cooperativity. There is a synergistic bond strengthening when a particular molecule acts simultaneously as an electron donor to one neighbor and donor to another. There is thus an energetic drive for the molecules in a finite cluster to arrange themselves in such a way as to maximize this cooperativity. The same driving force occurs within the crystal, but when the outer spheres of molecules are removed from the segment under consideration, the associated cooperativity is removed with them.

From a methodological point of view, the calculations demonstrate that an AIM bond path can signal the presence of a pnicogen bond, even if neither terminus of that path leads to a Pn atom. When the Pn-X bond, in this case Pn = As and X = F, is oriented so that it is not neatly aligned with the incoming electron donor, the associated σ*(PnX) antibonding orbital can be the sink for the charge being transferred, and the AIM bond path lead to X rather than to Pn. It is expected that this sort of finding is not unique to the AsF_3_ clusters considered here, but is a more general phenomenon applicable to other pnicogen bonds, and likely to other related σ-hole interactions as well, such as chalcogen and tetrel bonds. And in a more general sense, there have been a number of works that have documented reservations against an unquestioning acceptance of AIM bond paths and their interpretation [86,87,88,89,90,91,92,93,94,95,96].

It is commonly held that the three σ-holes generated around a PnX_3_ molecule would be conducive to the formation of up to three PnBs [97,98]. For example, the Cozzolino group [99] identified three such bonds in the internal geometry of Bi((NC_9_H_7_)_3_CH_3_) that persist in solution. A detailed examination of this question [100] provided a nuanced answer in the general case. The number of PnBs depends on both the nature of the base and Pn atom. First, in the case of an anionic nucleophile, PF_3_ and AsF_3_ can bond with only a single CN^−^, SbF_3_, and BiF_3_ can interact with two anions but only weakly. The weak NCH nucleophile can engage in a maximum of two PnBs, whereas three PnBs occur for NH_3_. This maximum can be extended to four PnBs but only for the heavier BiF_3_. Even then, the fourth PnB is somewhat longer and weaker than the others, and the entire (H_3_N)_4_···BiF_3_ complex relies partially on secondary interactions for its stability. A more recent work [101] showed that three intramolecular PnBs can occur as three covalently bonded −O(CH_2_)_n_X chains curve back on themselves, placing the basic X group in proximity to the central As or Sb.

The analysis of the clusters presented here has suggested that it is possible to form as many as five PnBs to a single Pn atom. However, not all of these five bonds are of full strength. Three of the five bonds are distorted from the optimal linear alignment of the nucleophile with the F-As bond extension, with θ(F-As··F) angles of less than 140°. Their cumulative NBO E2 perturbation energies are 1 kcal/mol or less, and ρ_BCP_ < 0.01 au. It also bears emphasis that these bonds do not occur within the context of an optimized geometry including only the central unit and its five neighbors. Rather, these arrangements are part of a far more extensive crystal geometry. Thus, although it is possible for the central As atom of AsF_3_ to engage in as many as five PnBs, this number comes with certain caveats.

The AsF_3_ molecule contains three well-defined and substantial σ-holes on the As atom, facilitated by the strong electron-withdrawing power of the F substituents. At the same time, the F atoms host strong negative potential regions. One would anticipate that the replacement of F by less electronegative substituents, like Cl or Br for example, would weaken the associated PnBs by reducing the magnitudes of both maxima and minima. On the other hand, replacement of As by its heavier and more polarizable congeners like Sb or Bi would have the opposite effect of magnifying both maxima and minima, thereby likely strengthening the connecting PnBs. Likewise, the transition from PnBs to other noncovalent bonds such as halogen or chalcogen bonds would alter both the number of σ-holes on each unit and the number of electron-donating agents. Future work will examine how such modifications affect the structure and bonding of both the crystal and the finite clusters.

## 5. Conclusions

The geometry of a finite fully optimized cluster is distinctly different than that of a segment within the crystal. Each molecule on the outer edge of a cluster orients itself so as to best interact with all of its neighbors that are present. In contrast, the molecules on the borderline of a finite segment of the crystal are disposed to interact not only with their neighbors on the border but also with those molecules that lie outside of the borderline. It is for this reason that the total interaction energy within an optimized cluster is much larger than that within a finite segment of the crystal. This distinction is present for all size clusters from 3 through 9. The average binding energy within an optimized cluster grows rapidly with *n* due to the growing number of bonds that are present and the cooperativity between them. Projection to an infinitely large cluster leads to average binding energy nearly three times that within a simple dimer. In contrast, consideration of a progressively larger segment of the crystal shows only a very modest gain with *n*, less than half that in the optimized cluster. Electron density topology analysis shows that in both the crystal and the fully optimized 14-mer, a single As atom is involved in five pnicogen bonds. Since each of the As atoms is associated with three σ-holes, such a case is an example of two bifurcated pnicogen bonds derived from a single atom.

In summary, then, a cluster of finite size will maximize attractive interactions between all of the molecules that are actually present within that aggregate. If this same group of molecules is placed within the context of an infinitely larger number of units, molecules that were on the periphery of the cluster will tend to reorient so as to accommodate the new units that are part of the larger crystal.

## Figures and Tables

**Table 1:** Characteristics of noncovalent bonds involving central molecule in nonamer.

Crystal			Cluster		
	R, Å	ρ, au		R, Å	ρ, au
As1–F7	2.890	0.0114	As5–F16	3.095	0.0085
As1–F20	2.990	0.0113	As5–F35	3.150	0.0082
As1–F18	3.202	0.0071	As5–F10	2.862	0.0125
As1–F32	3.182	0.0079	F6–As29	3.204	0.0064
As1–F26	3.437	0.0044	F6–F18	3.235	0.0033
F2–As9	3.202	0.0071	F7–As29	3.462	0.0044
F2–As33	3.437	0.0045	F7–As25	3.139	0.0078
F3–As21	2.890	0.0114	F8–As17	3.027	0.0096
F4–As9	2.990	0.0111			
F4–As13	3.182	0.0078			
sum		0.0839	sum		0.0607

**Table 2:** Sums of pairwise interaction energies.

	Crystal	Cluster
Σc-p ^a^	13.92 (15.3) ^b^	15.78
Σp-p	6.22	35.67
all pairs (sum of c-p and p-p)	20.14	51.45
actual E_int_	19.98	54.87

^a^ c—central unit, p—peripheral units. ^b^ E_int_ for c + p_8_; 15.1 if reoptimized central molecule.

**Table 3:** Characteristics of noncovalent bonds excluding central molecule in nonamer.

Crystal			Cluster		
	R, Å	ρ, au		R, Å	ρ, au
As21–F35	3.624	0.0040	As9–F15	3.086	0.0082
F11–As13	3.624	0.0039	F2–As13	2.968	0.0106
F12–F35	3.227	0.0030	As13–F22	3.062	0.0088
F14–F19	3.282	0.0023	F10–F16	3.103	0.0041
F14–F20	3.424	0.0018	As9–F20	3.153	0.0070
F15–F24	3.227	0.0030	F12–F20	2.915	0.0073
F20–F27	3.227	0.0030	As1–F20	3.157	0.0077
F22–F27	3.282	0.0022	F3–As21	3.112	0.0072
F22–F28	3.424	0.0018	F16–F23	2.923	0.0059
F26–F31	3.282	0.0023	F23–As33	3.035	0.0092
F26–F32	3.424	0.0019	As25–F36	3.189	0.0075
F31–As33	3.624	0.0040	As21–F26	2.988	0.0094
F7–As9	3.624	0.0040	F18–As29	3.421	0.0043
F7–F32	3.227	0.0032	F3–F30	3.201	0.0034
F8–F18	3.424	0.0018	F28–As29	3.068	0.0084
			As21–F30	3.098	0.0082
			F26–F30	3.019	0.0049
			As1–F32	3.141	0.0087
			F24–As33	3.419	0.0048
sum		0.0423	sum		0.1357

**Table 4:** Total and mean interaction energy for progressively larger size *n* of aggregates.

	−E_int_ (kcal/mol)		−E_int_/*n* (kcal/mol)	
*n*	Crystal	Cluster	Crystal	Cluster
2	3.13	4.44	1.57	2.22
3	4.82	10.00	1.61	3.33
4	7.06	16.95	1.77	4.24
5	9.25	24.73	1.85	4.95
6	11.52	31.55	1.92	5.26
7	16.06	40.14	2.29	5.73
8	17.97	46.49	2.25	5.81
9	19.98	54.87	2.22	6.10

**Table 5:** Geometric, AIM, and NBO properties of noncovalent bonds involving As and F of central molecule in 14-mer.

As1	R, Å	θ(F-As··F), degs	ρ, au	E2, kcal/mol
As1–F14	2.731	165.9	0.0167	4.47
As1–F47	2.921	163.5	0.0113	2.02
As1–F18	3.117	133.2	0.0082	1.04
As1–F27	3.207	139.6	0.0069	0.94
As1–F50	3.328	131.5	0.0061	0.49
F3				
F3–As33	2.975	166.8	0.0104	1.03
F3–As21	3.738	139.3	0.0030	0
F3–F26	2.914	-	0.0068	0.29
F3–F12	3.396	-	0.0024	0

## Data Availability

The data presented in this study are available on request from the corresponding authors.

## Sample Availability

Samples of the compounds are not available from the authors.
